# Effects of Two Types of Melatonin-Loaded Nanocapsules with Distinct Supramolecular Structures: Polymeric (NC) and Lipid-Core Nanocapsules (LNC) on Bovine Embryo Culture Model

**DOI:** 10.1371/journal.pone.0157561

**Published:** 2016-06-16

**Authors:** Eliza Rossi Komninou, Mariana Härter Remião, Caroline Gomes Lucas, William Borges Domingues, Andrea Cristina Basso, Denise Soledade Jornada, João Carlos Deschamps, Ruy Carlos Ruver Beck, Adriana Raffin Pohlmann, Vilceu Bordignon, Fabiana Kömmling Seixas, Vinicius Farias Campos, Silvia Stanisçuaski Guterres, Tiago Collares

**Affiliations:** 1 Programa de Pós-Graduação em Biotecnologia (PPGB), Grupo de Pesquisa em Oncologia Celular e Molecular, Biotecnologia/Centro de Desenvolvimento Tecnológico, Universidade Federal de Pelotas, Pelotas, 96010–900, RS, Brazil; 2 *In Vitro* Brasil S/A, Mogi Mirim, 13800–970, SP, Brazil; 3 Faculdade de Farmácia, Universidade Federal do Rio Grande do Sul, Av. Ipiranga, 2752, Porto Alegre, 90610–000, RS, Brazil; 4 Departamento de Química Orgânica, Instituto de Química, Universidade Federal do Rio Grande do Sul, Porto Alegre, 91501–970, RS, Brazil; 5 Department of Animal Science, McGill University, Sainte Anne de Bellevue, H9X 3V9, QC, Canada; Friedrich-Loeffler-Institute, GERMANY

## Abstract

Melatonin has been used as a supplement in culture medium to improve the efficiency of *in vitro* produced mammalian embryos. Through its ability to scavenge toxic oxygen derivatives and regulate cellular mRNA levels for antioxidant enzymes, this molecule has been shown to play a protective role against damage by free radicals, to which *in vitro* cultured embryos are exposed during early development. *In vivo* and *in vitro* studies have been performed showing that the use of nanocapsules as active substances carriers increases stability, bioavailability and biodistribution of drugs, such as melatonin, to the cells and tissues, improving their antioxidant properties. These properties can be modulated through the manipulation of formula composition, especially in relation to the supramolecular structures of the nanocapsule core and the surface area that greatly influences drug release mechanisms in biological environments. This study aimed to evaluate the effects of two types of melatonin-loaded nanocapsules with distinct supramolecular structures, polymeric (NC) and lipid-core (LNC) nanocapsules, on *in vitro* cultured bovine embryos. Embryonic development, apoptosis, reactive oxygen species (ROS) production, and mRNA levels of genes involved in cell apoptosis, ROS and cell pluripotency were evaluated after supplementation of culture medium with non-encapsulated melatonin (Mel), melatonin-loaded polymeric nanocapsules (Mel-NC) and melatonin-loaded lipid-core nanocapsules (Mel-LNC) at 10^−6^, 10^−9^, and 10^−12^ M drug concentrations. The highest hatching rate was observed in embryos treated with 10^−9^ M Mel-LNC. When compared to Mel and Mel-NC treatments at the same concentration (10^−9^ M), Mel-LNC increased embryo cell number, decreased cell apoptosis and ROS levels, down-regulated mRNA levels of *BAX*, *CASP3*, and *SHC1* genes, and up-regulated mRNA levels of *CAT* and *SOD2* genes. These findings indicate that nanoencapsulation with LNC increases the protective effects of melatonin against oxidative stress and cell apoptosis during *in vitro* embryo culture in bovine species.

## Introduction

*In vitro* culture conditions including the energy source, growth factors, pH, atmospheric oxygen concentration or transient light exposure, are associated with detrimental factors to the embryonic development, caused by enhanced levels of reactive oxygen species (ROS) [1]. These molecules are highly reactive and can oxidize DNA, proteins, and lipids resulting in mitochondrial alterations, ATP depletion, embryonic developmental arrest, low blastocyst production and defective embryo development [1, 2]. Additionally, ROS participate in cell signaling and modulation of cell death via caspase activation and regulation of anti-apoptotic and pro-apoptotic proteins of the Bcl-2 family [3].

Melatonin and its metabolites scavenge ROS and reduce the oxidative injury, promoting the development of oocytes and enhancing the quality of *in vitro* developed embryos in several species [4–14]. This molecule works through a myriad of signaling cascades that are protective to cells, acting dependently and independently of melatonin receptors (MT1 and MT2) to reduce free-radical formation [15]. The direct scavenger activity against toxic oxygen derivatives, and the ability to stimulate detoxifying enzymes like superoxide dismutase and glutathione peroxidase, have been described as the main melatonin mechanisms to intercept and prevent ROS production in embryos [16, 17].

Although melatonin easily crosses morphophysiological barriers, reaching cells and subcellular compartments in the organism [18], this molecule has an amphiphilic characteristic and is poorly soluble in aqueous solutions. These features can potentially limit its half-life, bioavailability and distribution into the cells depending on the biological environment [19]. *In vivo* and *in vitro* experiments have shown improved antioxidant and anti-apoptotic effects of melatonin against lipid peroxidation through its association with nanoparticulated systems (polymeric nanocapsules and solid lipid nanoparticles) in comparison to immediate release formulations [18–25].

Biocompatible engineered nanomaterials, especially nanoparticulated systems (NPs), has attracted the interest of many research groups due to its high loading capacity, stability and selective affinity that can represent a potential tool for delivering molecules into gametes and embryos [26, 27]. These systems that include solid lipid nanoparticles, polymeric nanocapsules and nanospheres [28], have been defined as colloidal particles having an average hydrodynamic diameter between 100 and 300 nm [29–31]. Within the NPs, nanoencapsulation has the advantage of entrapping the drug into the core, solubilizing of water insoluble drugs, and conferring drug protection against photochemical, chemical, or enzymatic degradation, improving the drug stability and efficacy [30, 32].

The materials that are used in surface and core of nanocapsules define its supramolecular structure. Distinct mechanical properties confer different advantages to the use of nanocapsules, like increasing of drug efficiency and reducing of toxicity and tissue irritation [30, 31, 33, 34]. Lipid-core nanocapsules (LNC), a new class of nanocapsules, have been shown to have some advantages over the polymeric nanocapsules (NC) [35]. Whereas the NC are composed of a liquid oily core surrounded by a polymeric nanometric film [23], LNC is formed of an organogel of sorbitan monostearate and capric/caprylic triglyceride, surrounded by poly(ε-caprolactone), and stabilized with polysorbate 80 micelles [22, 23, 31, 33, 34]. This organogel-structured core influenced the polymer wall structure making the LNC stiffer than NC [35]. Furthermore, the sorbitan monostearate dispersed in the oily-core interacts with the drug increasing the loading capacity more than 40 times compared to conventional nanocapsules [36].

Considering that *i*) melatonin loaded-Eudragit^®^ S100 nanocapsules (Mel-NC) can improve the antioxidant effects of melatonin, when compared to the non-encapsulated melatonin, that *ii*) LNC are promising intracellular carriers to melatonin, and that *iii*) no previous studies have been conducted to evaluate the effects of melatonin associated with nanoparticulated systems on cultured embryos, in this study, we examined the effects of melatonin associated with NC or LNC on *in vitro* development of bovine embryos.

## Materials and Methods

### Preparation of polymeric nanocapsules and lipid-core nanocapsules

Melatonin-loaded Eudragit^®^ S100 [poly(methacrylic acid-co-methyl methacrylate)] nanocapsules (Mel-NC; Röhm GmbH, Sontheim an der Brenz, Baden-Württemberg, Germany) [22] and melatonin-loaded lipid-core nanocapsules (Mel-LNC) were prepared by interfacial deposition of polymer [34, 37–39].

Mel-NC were prepared as previously reported using the self-assembly method [20]. Briefly, the acetone solution (63 mL) containing 12.5 mg melatonin (Sigma-Aldrich Co., St. Louis, Missouri, USA), 0.8 mL capric/caprylic triglyceride (Delaware, Porto Alegre, Rio Grande do Sul, Brazil), 250 mg Eudragit^®^ S100, and 192.5 mg sorbitan monooleate (Span 60; Sigma-Aldrich Co.) was added to an aqueous solution (125 mL) containing 192.5 g polysorbate 80 (Tween 80^®^; Delaware). The solvent was evaporated to eliminate the acetone and concentrate the turbid liquid solution to a final volume of 25 mL (0.5 mg/mL melatonin).

Mel-LNC were prepared by using the self-assembly general method described for LNC [34, 38, 40]. Briefly, 250 mg poly(ε-caprolactone) (PCL; Mn = 80 kg L^-1^); Sigma-Aldrich Co.), 95 mg sorbitan monostearate (Sigma-Aldrich Co.), 0.404 mL capric/caprylic triglyceride and 12.5 mg melatonin were dissolved in 63 mL acetone at 40°C. This organic phase was injected into an aqueous phase containing 192.5 mg polysorbate 80 micelles dispersed in 125 mL water at 40°C. A turbid solution was obtained instantaneously and kept under magnetic stirring for 10 minutes. Acetone was then eliminated by evaporation, and the liquid turbid solution was concentrated under reduced pressure at 40°C. The final volume was adjusted to 25 mL in a volumetric flask (0.5 mg/mL melatonin).

Drug-unloaded polymeric nanocapsules (NC) and lipid-core nanocapsules (LNC) formulations were prepared as described above but without the addition of melatonin. Due to its low aqueous solubility, non-encapsulated melatonin (Mel; 0.5 mg/mL) was prepared in sterile water containing 0.5% dimethyl sulphoxide (DMSO; Sigma-Aldrich Co.). In contrast, it was not necessary to solubilize the NC, LNC, Mel-NC and Mel-LNC formulations in DMSO.

### Particle size distribution and specific surface

The volume weighted average diameter (D_4,3_), the polydispersity (expressed as *SPAN*) and the specific surface area of the nanocapsules were determined by laser diffraction [41] using a Mastersizer^®^ 2000 instrument (Malvern Instruments, Malvern, Worcestershire, England). Each nanocapsule suspension was directly poured into the wet dispersion unit containing about 100 mL of distilled water, in quantity enough to reach an obscuration between 0.02 and 0.10. The size distribution profiles were obtained discounting the background signal of distilled water (dispersion medium). The D_4,3_ calculated by the Mie theory corresponds to the geometric diameters for spherical particles, and *SPAN* was calculated using the follow equation:

$$SPAN=\frac{d_{90}-d_{10}}{d_{50}}$$

where *d*_*10*_, *d*_*50*_ and *d*_*90*_ are the diameters under the size distribution curve at percentiles 10, 50 and 90. The diameter at percentile 50 by number (median) of particles (*d*_*50*,*n*_) was also calculated.

### Drug content

Drug content (*C*_*total*_) was assayed by HPLC (high-performance liquid chromatography) using a Perkin Elmer Series 200 chromatograph and Lichrospher RP-18 column (5 μm; 250 × 4 mm; Merck, Darmstadt, Hessen, Germany). The mobile phase was composed of acetonitrile/water (55:45, v/v). Melatonin was detected at 229 nm. This method was previously validated [22] considering linearity (*R*^2^ = 0.9998 for 2.5 to 17.5 mg/mL), intermediate precision (relative standard deviation = 3.1%), repeatability (relative standard deviation = 1.4 to 1.8%) and accuracy (101±1%). Drug content (*C*_*total*_) was determined after the dissolution of Mel-NC and Mel-LNC in acetonitrile, dilution in the mobile phase, filtration (0.45 μm, PVDF, Millipore Millex—HV) and injection in the chromatograph.

### Oocyte recovery and *in vitro* maturation (IVM)

Ovaries of Aberdeen and Red Angus (*Bos taurus*) heifers, ranging from 18 to 36 months of age, were collected from a local slaughterhouse (Pelotas, Rio Grande do Sul, Brazil). Cumulus-oocytes complexes (COCs) were aspirated from 2–8 mm follicles using a sterile 18-gauge needle attached to a disposable syringe. Only oocytes with homogeneous cytoplasm and a minimum of two layers of compact cumulus cells were selected. Groups of 15–20 COCs were matured in 90 μL of IVM medium, under mineral oil at 38.5°C and 5% CO_2_ for 22–24 hours. The IVM medium was composed of TCM-199 (Gibco^®^, Thermo Fisher Scientific inc., Waltham, Massachusetts, USA) supplemented with 10% FCS (Gibco^®^, Thermo Fisher Scientific inc.), 1 μg/mL FSH (*Folltropin-*V^®^; *Bioniche Animal Health*, Belleville, Ontario, Canada), 50 UI/mL hCG (Profasi, Serono, Sao Paulo, Brazil), 1 μg/mL estradiol (17β-estradiol; Sigma-Aldrich Co.), 0.2 mM sodium pyruvate (Sigma-Aldrich Co.), and 83.4 μg/mL amikacin (Sigma-Aldrich Co.).

### *In vitro* fertilization (IVF)

After IVM, the COCs were transferred in groups of 20–25 to 90 μL drops of IVF medium composed of TALP-IVF medium, with 0.2 mM sodium pyruvate, 83.4 g/mL amikacin, and 0.6% bovine serum albumin (BSA; Sigma-Aldrich Co.), supplemented with 20 μg/mL heparin and 80 μg/mL, penicillamine, hypotaurine, and epinephrine (PHE) solution. Cryopreserved semen straws from an Aberdeen Angus bull were thawed for 30 s in a water bath at 35°C. Sperm was washed by two centrifugation steps at 200 × *g* for 5 minutes: in the first step it was washed in 2mL of TALP medium, with 0.2 mM sodium pyruvate, 83.4 g/mL amikacin, that was supplemented with 10 mM HEPES (Sigma-Aldrich Co.), and in the second washing in 2 mL of TALP-IVF medium. After visual assessment of motility, spermatozoa concentration was adjusted to 25 × 10^6^ live sperm/mL, and each fertilization drop received 4μl of sperm (final concentration of 1×10^5^ sperm per drop).

### *In vitro* culture (IVC)

Eighteen hours after IVF, the presumptive zygotes were stripped of cumulus cells using a pipette and then washed in a modified synthetic oviduct fluid culture medium (SOFaa BSA, *In Vitro* Brasil S/A, Mogi Mirim, Brazil). Selected zygotes with intact ooplasmic membrane were randomly allocated to experimental groups and cultured for 7 days in 100 μL droplets of SOFaa BSA at 38.5°C and 5% CO_2_ in air (approximately 20% of O_2_). At 72 and 120 hours of culture, two feedings were performed by replacing 50% of the culture medium of each drop with fresh medium maintaining the same initial concentration for each treatment. The medium used in the second feeding was supplemented with1μg/mL D-(+)-Glucose (Sigma-Aldrich Co, Missouri, USA).

### Serial dilution of the formulations and supplementation in the culture medium

The initial drug concentration in the stock formulations of Mel-NC, Mel and Mel-LNC, was ~ 2 × 10^-3^M. The concentration of the stock formulations was then adjusted to 1 × 10^-3^M by 1:1 dilution in milli-Q filtered water. Stock solutions (100 x) of working concentrations (1 × 10^-6^M, 1 × 10^-9^M, and 1 × 10^-12^M) were prepared by serial dilutions in milli-Q filtered water. Working solutions were prepared by diluting 1 μL of the stock solution in 99 μL of SOFaa BSA. For unloaded NC and LNC controls groups, equivalent volumes used to dilute the highest concentration of the nanocapsule groups containing melatonin (1 × 10^-6^M) were used. The concentration of nanocapsules in the stock formulations was estimated using a flow-cytometer (Guava^®^ Flow Cytometry easyCyte™ System, Merck KGaA, Darmstadt, Germany). Values for Mel-NC, NC, Mel-LNC and LNC in the stock formulations were 2843, 2979, 2916 and 1754 nanocapsules/μL, respectively.

### Embryo evaluation

The proportion of oocytes that developed to 2- (cleaved), 4-, 8-, 16-cell, morulae and blastocyst stages were determined in nine replicates. At the end of the experiment, a total of 1839 presumptive zygotes allocated in twelve experimental groups (~ 150 per group) were assessed from day 1 to day 7 of culture by visual observation in a stereoscope. In five of these replicates, embryos that developed to the blastocyst stage were kept in culture until day 9 to evaluate hatching rates. Blastocysts produced in the other four replicates were fixed at day 7 to determine the total number of cells and the rate of cell apoptosis by terminal deoxynucleotidyl transferase dUTP nick end labeling (TUNEL) assay.

### Detection of cell apoptosis by TUNEL assay

DNA fragmentation was analyzed using a simultaneous nuclear staining and TUNEL assay protocol, following the manufacturer’s instructions with minor modifications. For TUNEL preparation, expanded blastocysts at day 7 of culture were fixed for 1 hour at room temperature in 4% paraformaldehyde. Fixed embryos were washed in 70 μL PBS-PVP solution, permeabilized with 0.5% Triton X-100 in PBS for 30 minutes at room temperature in a humidified box, and then washed again in 3 drops of PBS-PVP solution. Positive control embryos, from control group, were treated with 50 μL of DNase I solution [3 U/mL DNase I (Invitrogen^™^, Thermo Fisher Scientific inc., Waltham, Massachusetts, USA) in 50 mM Tris-HCl, pH 7.5] for 20 minutes at 37°C, and then washed in PBS-PVP before proceeding with TUNEL labeling. Embryos were then incubated in fluorescein-conjugated dUTP and TdT (In Situ Cell Death Detection Kit, Fluorescein; Roche Diagnostics, Mannheim, Baden-Württemberg, Germany) for 1 hour at 38.5°C and 5% CO_2_. Negative control embryos, from control group, were incubated in the labeling mix without TdT. After the reaction was stopped, embryos were washed in PBS-PVP and transferred into 10 mg/mL Hoechst 33342 (Sigma-Aldrich Co.) in PBS for 30 minutes at room temperature in the dark. Embryos were then washed three times in PBS-PVP and mounted on slides and cover slips. Fluorescence emissions were recorded using a digital camera DP72 (Olympus Corporation, Shinjuku-ku, Tokyo, Japan) attached to an inverted fluorescent microscope IX 71 (Olympus Co.). The recorded fluorescent images were analyzed using the Cell^F software (Olympus SIS-Soft Imaging Solutions, Münster, North Rhine-Westphalia, Germany). Total number of nuclei and nuclei with fragmented DNA were evaluated in each embryo. Apoptotic cell rate (DNA-fragmented nucleus index) was calculated by dividing the number of cells with fragmented DNA by the total cell number [42]. The experiment was performed in four replicates with 3–4 embryos per group from each replicate.

### Measurement of Reactive Oxygen Species (ROS) levels

ROS levels in embryos were measured by 2',7'-dichlorodihydrofluorescein diacetate (DCHFDA; Sigma-Aldrich Co.) according to the modified protocol previously described [2, 43, 44]. The measurement was performed at day 3 post-fertilization in non-fragmented embryos at 4–8 cell-stage. The DCHFDA was freshly prepared in DMSO at 1 × 10^−3^ M before each experiment, kept in the dark, and used up to 48 hours after preparation. Embryos were incubated in IVC medium containing 1 μM DCHFDA for 20 minutes at 38.5°C and then washed in IVC medium before being placed on a plate. For positive control, oocytes from control group were previously incubated for 1 hour in 50μM H_2_O_2_. Fluorescence emissions were recorded using a digital camera DP72 attached to an inverted fluorescent microscope IX 71 (Olympus Co.) after excitation at 480 nm and emission at 510 nm. The recorded fluorescent images were analyzed using the Cell^F software (Olympus SIS-Soft Imaging Solutions, Münster, North Rhine-Westphalia, Germany). Eight points per embryo were marked in each fluorescent image, and the average pixel intensity per embryo was used to compare ROS production among different groups. The experiment was performed in triplicate with 6–8 embryos per group in each replicate.

### RNA extraction, cDNA synthesis, and Real-Time PCR

Three pools of 8 blastocysts were prepared from each group after evaluating the hatching rate at day 9 of culture. Embryos were washed twice in PBS-PVP solution (1 μg/mL polyvinylpyrrolidone in PBS), snap frozen and stored at -80°C for subsequent RNA extraction. Poly (A) RNA was isolated using the Dynabeads^®^ mRNA DIRECT^™^ Micro Kit (Thermo Fisher Scientific inc., Waltham, Massachusetts, USA) following the manufacturer’s instructions with minor modifications, as previously described [45, 46]. Reverse transcription was conducted using the High Capacity cDNA Reverse Transcription Kit (Thermo Fisher Scientific inc.), according to manufacturer’s instructions, and relative levels of each transcript were calculated by normalization to the abundance of β-actin as the internal control. Reactions were run on a Stratagene^®^ Mx3005P^™^ Real-Time PCR System [Agilent Technologies, Santa Clara, California, USA, using SYBR^®^ Green PCR Master Mix (SYBR^®^ Green PCR Master Mix, Thermo Fisher Scientific inc.)]. PCR was performed by adding 2 μL of samples to the PCR mix containing specific primers for each gene. Primer sequences and annealing temperatures for all transcripts are shown in S1 Table. Amplification was carried out at cycling conditions of 95°C for 2 minutes, followed by 40 cycles of at 95°C for 15 seconds, 55–60°C for 60 seconds. Changes in the relative gene expression of the target were determined using the formula: 2–[Delta][Delta]Ct [47]. PCR runs for each cDNA sample were performed in duplicate.

### Experimental design

The first experiment was designed to evaluate the effect of different concentrations of melatonin alone or loaded in nanocapsules (NC) or lipid-core nanocapsules (LNC). Melatonin concentrations tested in this experiment (10^−6^, 10^−9^, and 10^−12^ M) were based on previous studies which evaluated the effects of non-encapsulated melatonin on *in vitro* maturation of oocytes, *in vitro* fertilization and *in vitro* embryo development in porcine [2, 12, 47], bovine [4, 6], and mice [13, 48].

Based on the results of embryo development and hatching obtained in the first experiment, the 10^−9^ M concentration of melatonin was chosen to be used in the subsequent experiments to investigate the effect of the nanocapsule type (NC vs. LNC) on embryo cell number, cell apoptosis, ROS production in 4–8 cells stage embryos and relative mRNA abundance of genes in blastocysts. For these experiments, embryos were cultured in control media without melatonin, in the presence of 10^−9^ M melatonin alone (Mel) or loaded in nanocapsules (Mel-NC or Mel-LNC).

### Statistical analyses

Chi-square analysis was performed to compare cleavage, blastocyst formation, and hatching rates. Total cell number, apoptosis, ROS levels, and mRNA levels of genes in embryos were compared among experimental groups using one-way analysis of variance (ANOVA) followed by Tukey’s test for multiple comparisons. Transcript levels of the PRDX5 gene were compared by Kruskal-Wallis one-way ANOVA test when variances were unequal. The results were reported as the mean values for each set of data ± SEM and the degree of statistical significance in all analyses was defined at a probability level of *P< 0*.*05*.

## Results

### Nanocapsules evaluation

Formulations were produced as homogeneous white milky liquids. The volume weighted average diameters (D_4,3_) of the nanocapsules were 237 nm and 288 nm (Mel-NC and NC, respectively) while the values for the lipid-core nanocapsules were 171 nm and 205 nm (Mel-LNC and LNC, respectively). The polydispersity of their size distribution profiles was respectively 2.18, 2.54, 1.7 and 1.9, whereas the specific surface areas were 45, 41, 47 and 41 m^2^ g^-1^, and the d_*50*,*n*_ were 64, 65, 64 and 65 nm, respectively. Melatonin content was 0.49 mg/mL in Mel-NC and 0.41 mg/mL in Mel-LNC.

### Effects of free and nanoencapsulated melatonin on embryo development

**Fig 1:**
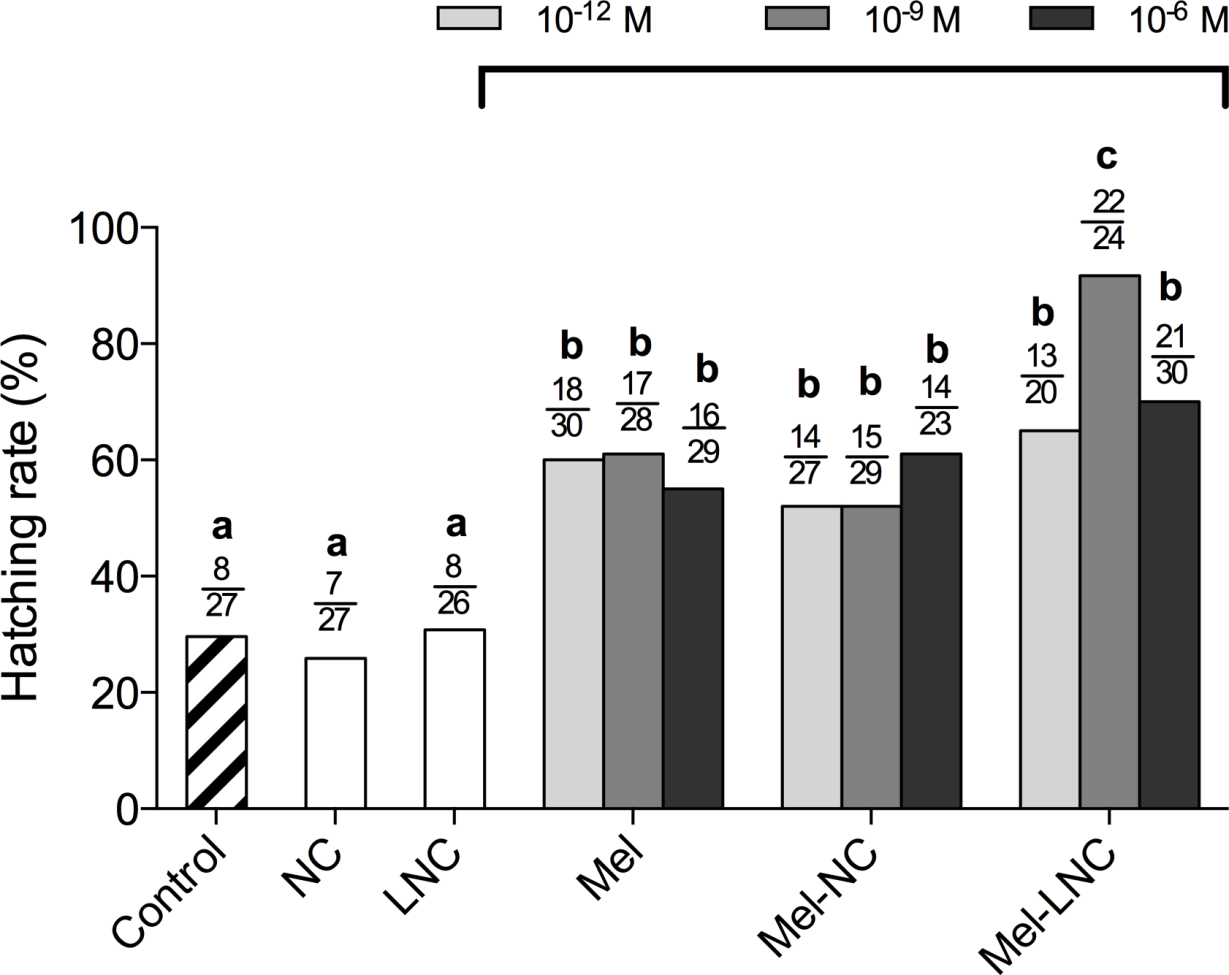
Hatching rates of bovine embryos cultured in the presence of free or nanoencapsulated melatonin. Mel = Non-encapsulated melatonin, Mel-NC = melatonin-loaded polymeric nanocapsules, Mel-LNC = melatonin-loaded lipid-core nanocapsules, NC = drug-unloaded nanocapsules, LNC = drug-unloaded lipid-core nanocapsules. Melatonin was used at 10^−12^ M, 10^−9^ M or 10^−6^ M concentrations. Control group was cultured in SOFaa BSA alone. The numerators represent the numbers of hatched blastocysts and the denominators represent total blastocysts in each group. Different letters (a-c) above the bars indicate significant differences between groups (*P* < 0.05).

There was no difference between treatments on cleavage rates or in the proportion of oocytes that developed to 4-, 8-, 16-cell, morulae and blastocyst stages (*P*> 0.05, S2 Table). However, the rates of hatched blastocysts in the Mel, Mel-NC, and Mel-LNC-treated groups were higher (*P< 0*.*05*, Fig 1 and S3 Table) than in the control groups (Control, NC and LNC). In addition, Mel-LNC at the 10^−9^ M concentration produced the highest hatching rate (92%) in comparison with all other treatments (*P< 0*.*05*, Fig 1 and S3 Table).

### Effects of free and nanoencapsulated melatonin on cell apoptosis in blastocysts

**Fig 2:**
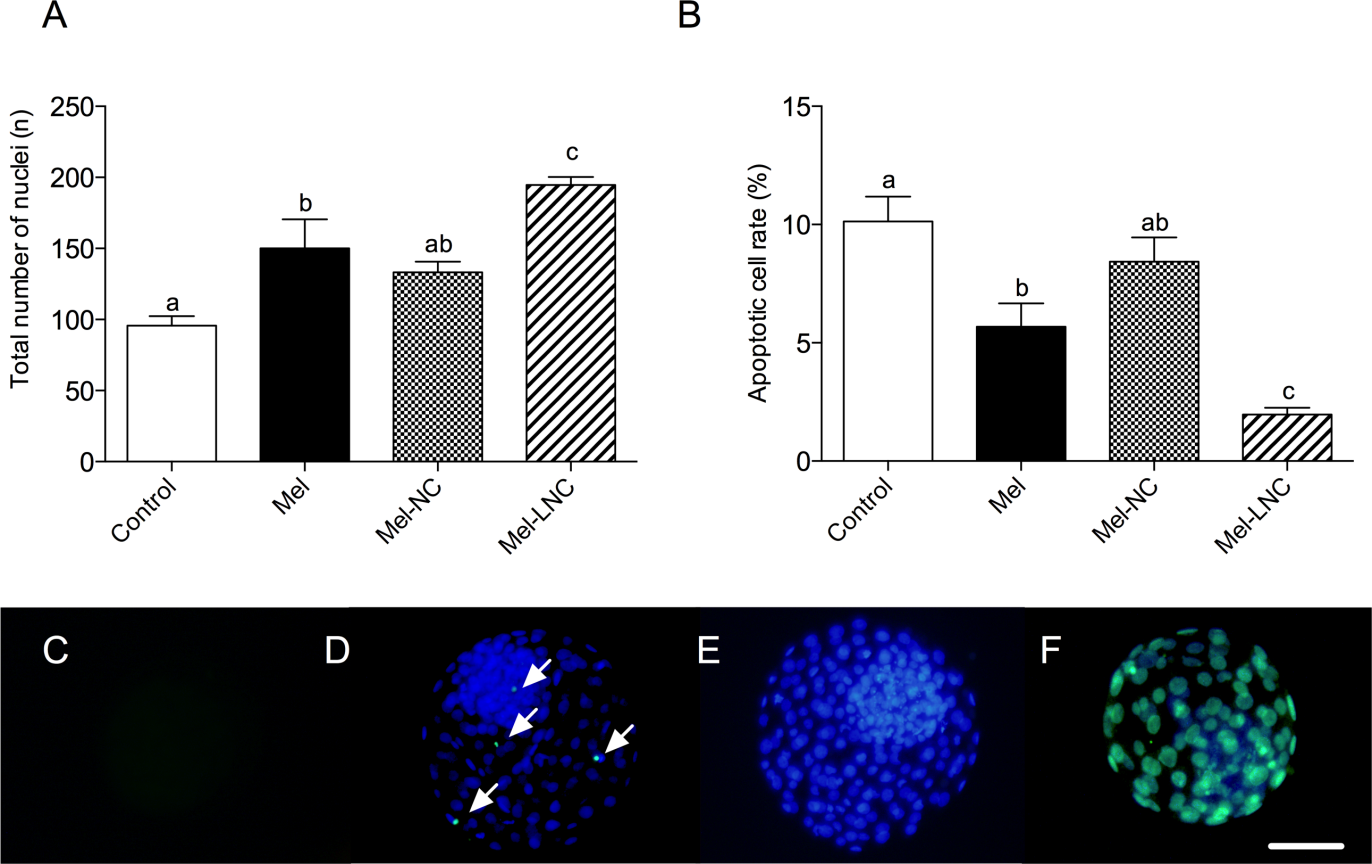
Effects of free and nanoencapsulated melatonin (10^−9^ M) on total cell number and cell apoptosis in blastocysts. Effect of treatment on total cell number (A), and apoptotic cell rate in blastocysts (B). Data represent mean ± S.E.M. Different letters above the error bars indicate significant differences between groups (P< 0.05). Representative images of negative control (C), apoptotic cells indicated by arrows (D), total cell number (E), and positive control embryo with both nuclear and TUNEL staining (F). Scale bar = 50 μm. Magnification = 100X.

The total cell number in blastocysts from the Mel-treated group was higher than in the control group (*P< 0*.*05*), but not different (*P> 0*.*05*) from the Mel-NC-treated group (Fig 2A and S4 Table). The highest number of cells was observed in embryos treated with Mel-LNC at 10^−9^ M, which was significantly superior (*P< 0*.*05*) to all other groups (Fig 2A and S4 Table). The apoptosis rate was lower in the Mel-treated group than control group (*P< 0*.*05*), but not different (*P> 0*.*05*) from the Mel-NC-treated group (Fig 2B and S4 Table). Embryos treated with Mel-LNC had the lowest apoptotic cell rate (*P*< 0.05) among all treatments (Fig 2B and S4 Table).

### Effects of free and nanoencapsulated melatonin on embryo ROS levels

**Fig 3:**
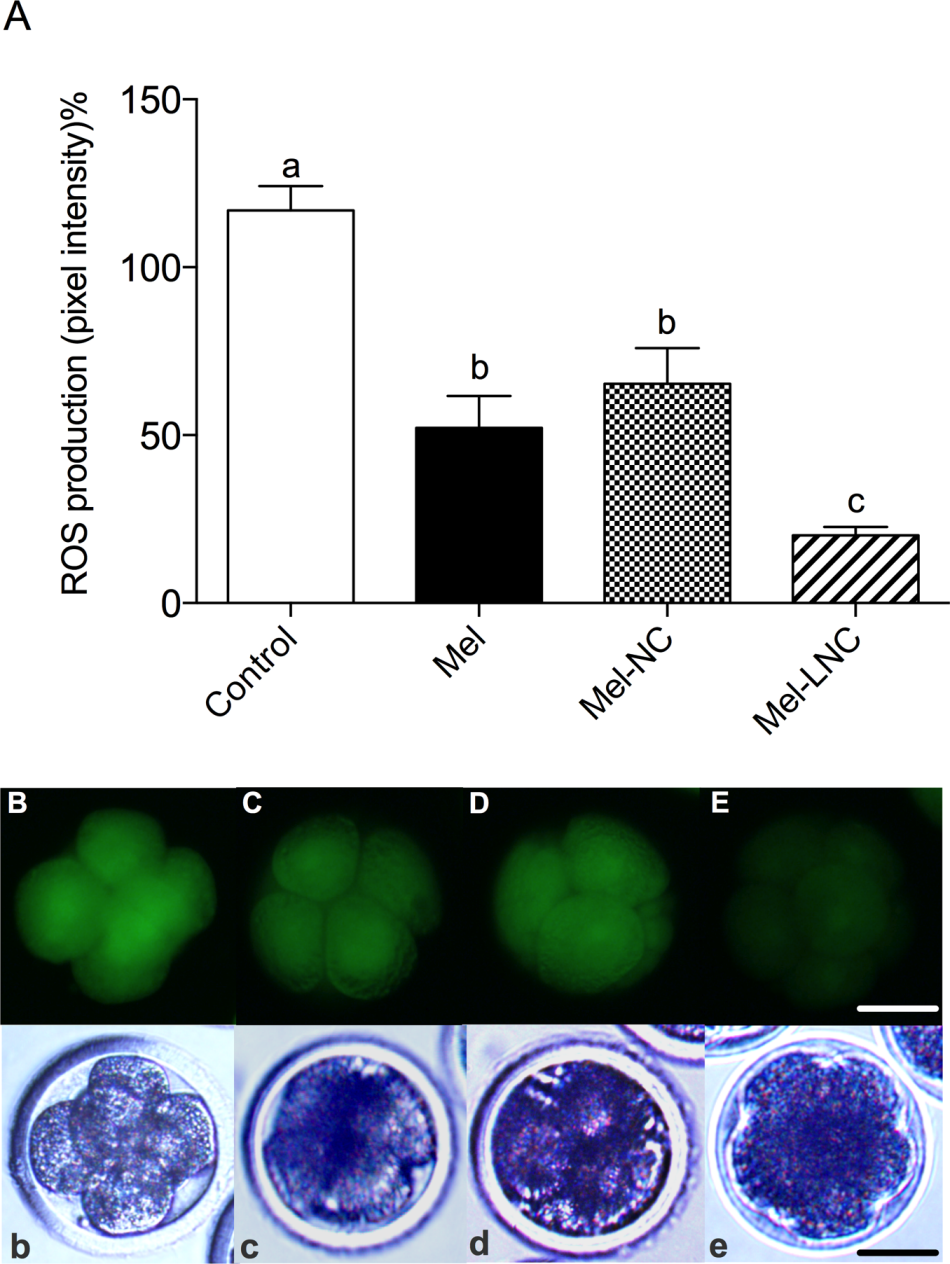
ROS levels in 4–8 cell stage embryos cultured in the presence of free and nanoencapsulated melatonin. Embryos were cultured in the presence of 10^−9^ M melatonin non-encapsulated (Mel) or encapsulated in nanocapsules (Mel-NC) or lipid-core nanocapsules (Mel-LNC). In the control group embryos were cultured in SOFaa BSA medium alone. Data represent mean ± S.E.M. Different letters above the error bars indicate significant differences between groups (P< 0.05). Representative fluorescent (B, C, D, E) and corresponding bright field (b, c, d, e) images of control, Mel, Mel-NC, and Mel-LNC embryos, respectively. Scale bar = 50 μm. Magnification = 100X.

The ROS levels in 4–8-cell embryos were lower (*P*< 0.05) in the Mel-treated group than in the control group (Fig 3A and 3B), but not different (*P*> 0.05) from the Mel-NC-treated group (Fig 3A and 3D). The ROS levels in Mel-NC-treated embryos were lower (*P*< 0.05) than control group (Fig 3A and 3C). Embryos treated with Mel-LNC had the lowest ROS levels (*P*< 0.05) among all treatments (Fig 3A and 3E).

### Effects of free and nanoencapsulated melatonin on the relative mRNA abundance of genes in blastocysts

**Fig 4:**
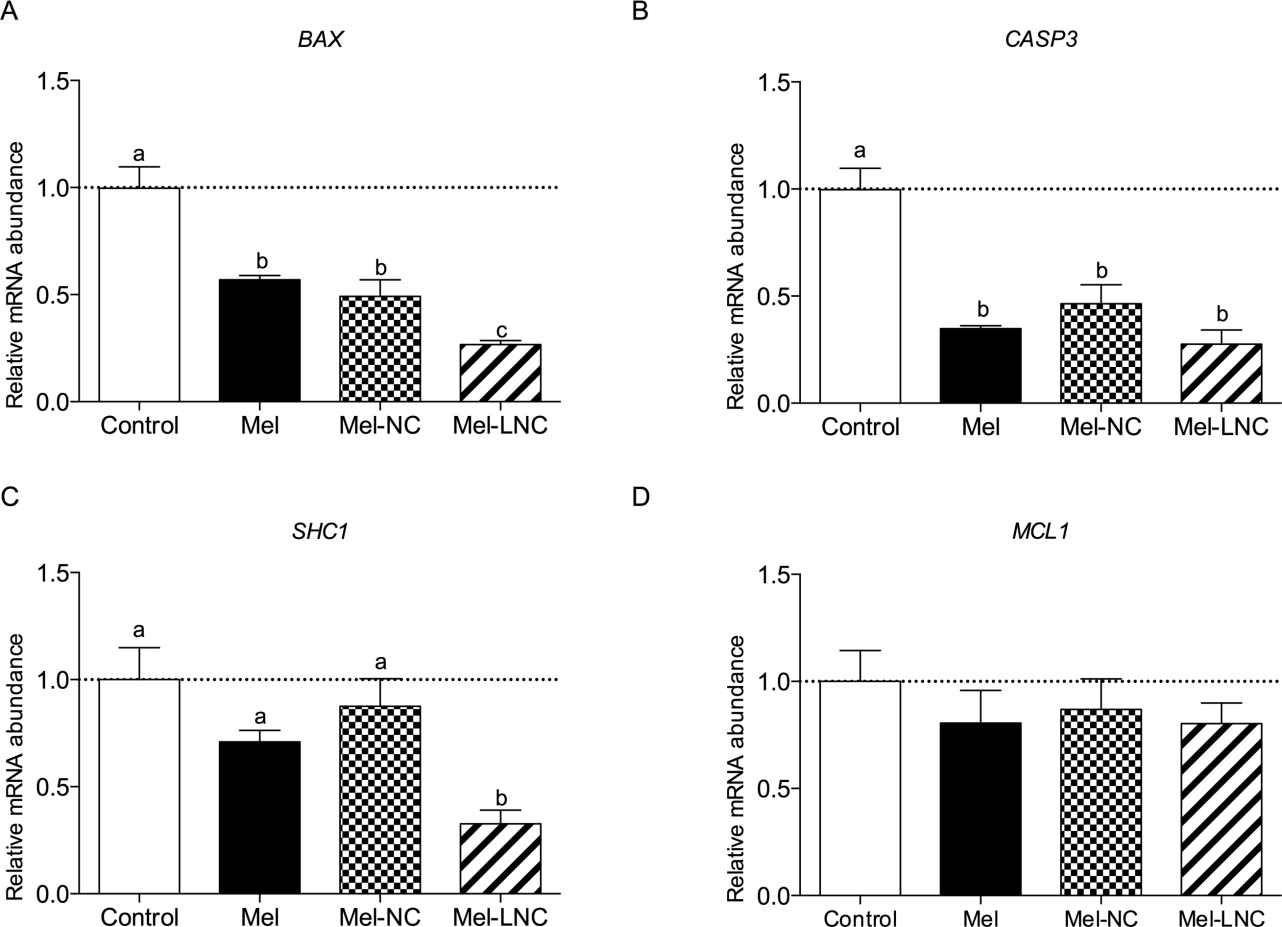
Effects of free and nanoencapsulated melatonin (10^−9^ M) on the relative mRNA abundance of apoptosis-related genes. Transcript levels of four apoptosis-related genes BAX (A), CASP3 (B), SHC1 (C) and MCL1 (D) were quantified by q-PCR. Data represent mean ± S.E.M. Different letters (a, b and c) indicate significant differences between groups (P< 0.05).

Transcript levels of the pro-apoptotic *BCL2*-associated X protein (*BAX*) gene were lower in blastocysts treated with Mel and Mel-NC compared to control embryos (*P<* 0.05). Blastocysts from the Mel-LNC group had the lowest *BAX* mRNA levels among all treatments (Fig 4A, *P< 0*.*05*). Transcript levels for the apoptosis-related cysteine peptidase 3 (*CASP*3) gene, were lower in blastocysts from Mel, Mel-NC and Mel-LNC groups compared to the control group (Fig 4B, *P*< 0.05). The abundance of mRNA for the SHC-transforming protein 1 *(SHC1*) gene was reduced only in embryos from the Mel-LNC group (Fig 4C, *P<* 0.05). The transcript levels for the myeloid cell leukemia sequence 1 (*MCL1*) gene were not significantly different among treatments (Fig 4D, *P*> 0.05).

**Fig 5:**
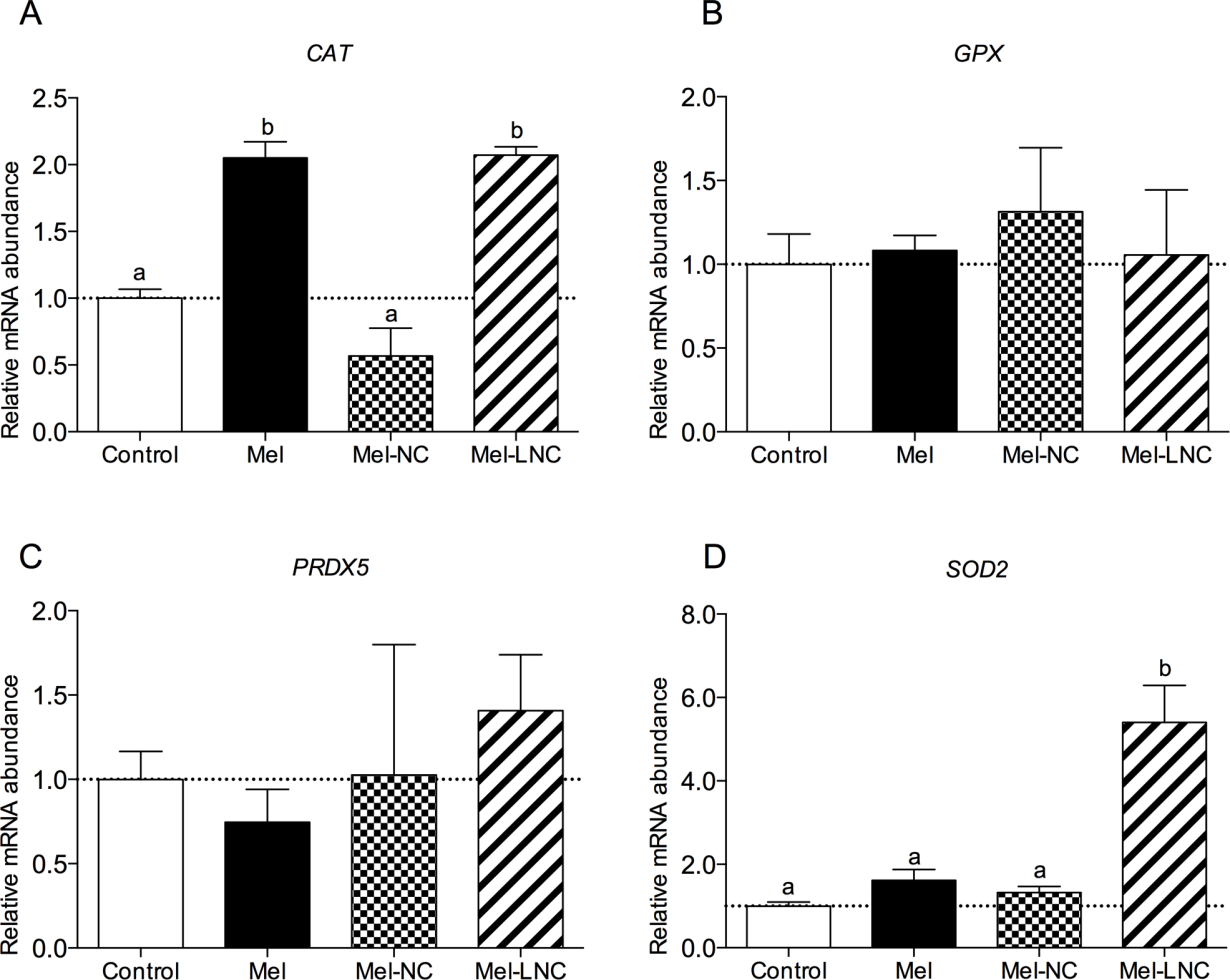
Effects of free and nanoencapsulated melatonin (10^−9^ M) on the relative mRNA abundance of oxidative stress-related genes. Data represent mean ± S.E.M. Different letters (a, b and c) indicate significant differences between groups (P< 0.05).

Transcript levels for the oxidative stress-related catalase (*CAT*) gene were higher in blastocysts from the Mel and Mel-LNC groups (*P*< 0.05) in comparison to control and Mel-NC groups (Fig 5A). Messenger RNA abundance for the glutathione peroxidase (*GPX*) and peroxide redoxin (*PRDX5)* genes were similar in embryos from all the treatments (Fig 5B and 5C *P*> 0.05). Transcript levels for the superoxide dismutase 2 (*SOD2)* gene were higher in blastocysts from the Mel-LNC group compared to control, Mel and Mel-NC groups (*P*< 0.05, Fig 5D).

**Fig 6:**
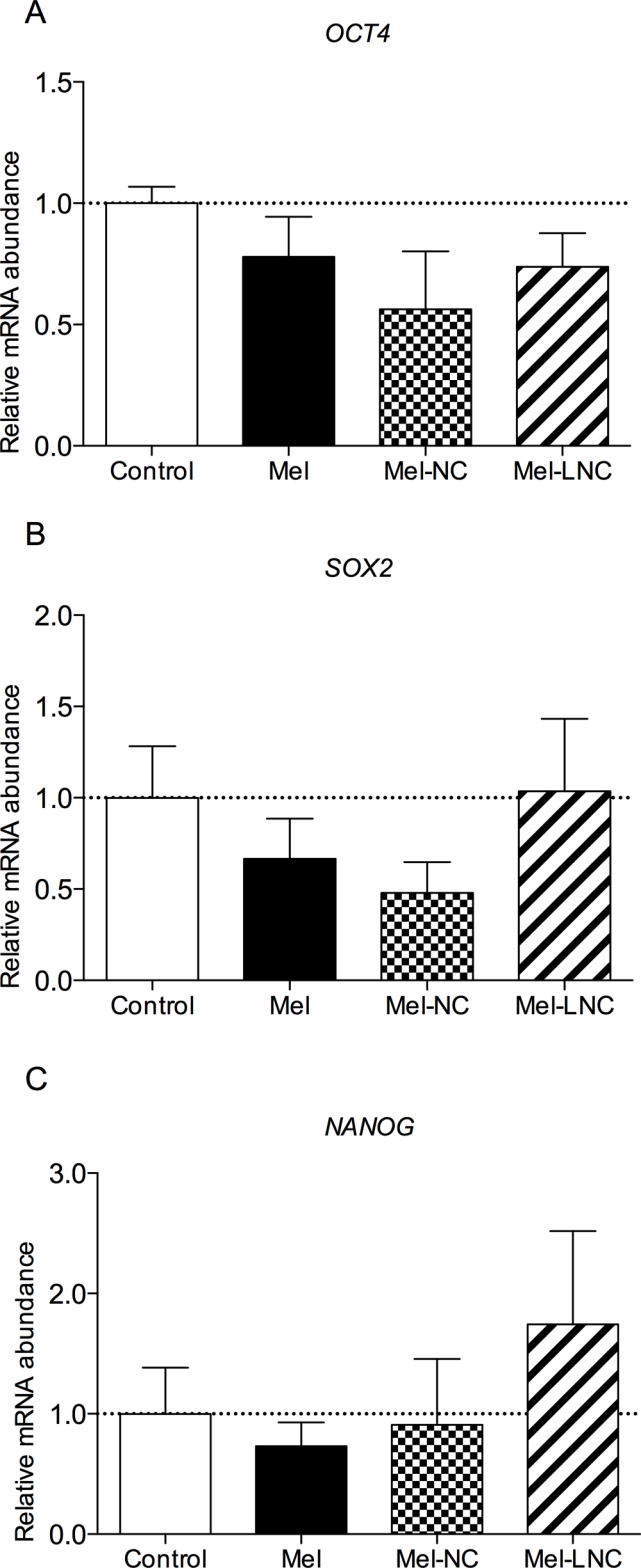
Effects of free and nanoencapsulated melatonin (10^−9^ M) on the relative mRNA abundance of pluripotency-related genes in bovine blastocysts. Data represent mean ± S.E.M.

The relative abundance of mRNA for the pluripotency-related genes POU class 5 homeobox 1 (*OCT4)*, SRY (sex determining region Y)-box 2 (*SOX2)* and nanog homeobox (*NANOG)* genes was not different among embryos derived from the different treatments (Fig 6A, 6B and 6C, *P*>0.05).

## Discussion

Embryos derived from *in vitro* culture systems are exposed to a variety of potentially harmful factors as a result of suboptimal culture conditions that promote lower development rates in relation to *in vivo-*derived ones [49]. For this reason, the search for new components and a better use of elements in the available formulations of embryonic culture media are envisioned as a primary research area in embryology.

One important improvement in the culture medium is melatonin supplementation for embryo *in vitro* production. It has been shown to accelerate and enhance nuclear maturation rates [50], increase cumulus cells expansion, improve mitochondria distribution [51], decrease ROS levels [11, 51, 52], and improve developmental competence generating higher quality blastocysts by increasing the cell numbers per blastocyst [2, 10, 12, 47, 53] and increasing the hatching blastocyst rates [10].

However, melatonin has some limitations in relation to its chemical properties, such as low solubility in water, low bioavailability, and short biological half-life. To overcome these problems, sustained release dosage forms to deliver melatonin *in vivo* have been investigated since these formulations were reported to be clinically more useful when compared to immediate release formulations [24, 25]. In addition, the administration of melatonin in nanoparticulated systems *in vitro* have been demonstrating advantages in relation to non-encapsulated melatonin as with other molecules with similar shortcomings [25, 54–56].

The highest cell number and hatching rate were found in embryos derived from Mel-LNC group, suggesting that nanoencapsulation of melatonin may contribute to its bioavailability and intracellular delivery. The drug association with the carriers was similar, with 0.49 mg/mL melatonin content in Mel-NC and 0.41 mg/mL in Mel-LNC. We believe that 41 mg/mL in Mel-LNC can be more effective due to its mechanical properties, surface and core chemistry. In relation to the core, we demonstrated previously that the organogel formed the core of the LNC is a second diffusional barrier for the drug release [57]. In addition, the LNC have a more rigid core and polymer wall compared to polymeric nanocapsules prepared without sorbitan monostearate (the solid component of the core) [35].

Another factor is the variation in size distribution and mean particle diameter, due its impact on the surface area [38]. Generally, nanoparticles prepared by different methods have average diameters between 100 and 300 nm [29]. LNCs are stiffer and smaller than NCs [35]. The size of Mel-NC and Mel-LNC had average diameter D_4,3_ of 237 nm and 171 nm, respectively. Despite this slight difference, the smaller size of the Mel-LNC may have made it easier for passage through the biological barriers, providing better integration and delivery of melatonin to the embryonic subcellular compartments, such as mitochondria and nuclei. In addition, it is known that smaller particles with higher flexibility give deeper penetration in skin [35]. However, in oocytes and embryos, it is still unclear how nanomaterials can cross zona pellucida and plasmatic membrane, and if this occurs spontaneously or through specific channels [58–60].

In our study, we also observed that blastocysts treated with Mel-LNC exhibited apoptosis frequency significantly lower than all other groups. In addition, the relative abundance of mRNA for apoptosis showed that pro-apoptotic *BAX*, *CASP3*, and *SHC1* genes were down-regulated by Mel-LNC treatment. Apoptosis can be induced by oxidative stress caused by ROS accumulation during *in vitro* culture of embryos resulting in lower blastocyst rate, low quality, and increased number of apoptotic nuclei [2]. Wang and co-workers [10] reported that melatonin treatment up-regulates relative abundance of mRNA for anti-apoptotic factor *BCL2* while down-regulating abundance of mRNA for pro-apoptotic genes, *p53* and *CASP3*.

We showed that Mel and Mel-LNC were able to significantly reduce the ROS levels in 4–8 cell-stage embryos. However, embryos treated with Mel-LNC showed the lowest production of ROS. Although we did find that Mel-LNC reduce ROS levels, the abundance of mRNA for the *CAT* and *SOD2* genes were altered in different ways. The *CAT* transcript levels were up-regulated in blastocysts treated with Mel or Mel-LNC in comparison with the control and Mel-NC groups while *SOD2* transcript levels were up-regulated only in Mel-LNC group. These results may suggest that the ability of these embryos to respond to O_2_^-^ is impaired or, the steady-state level of O_2_^-^ is lower in the groups with low *SOD2* activity, even if the total oxidant concentration is elevated. In our study, *GPX* was not altered by treatment with both, Mel and Mel-LNC, even when transcript levels were up-regulated for the *SOD2* gene. Previous studies reported that melatonin up-regulates the expression of *SOD2* without affecting *GPX* [10].

Our result demonstrates that both Mel-NC as Mel-LNC did not induce aberrant expression of pluripotency genes, *OCT4*, *SOX2*, and *NANOG*, thus maintaining the pluripotency potential in bovine embryonic cells. These genes are associated with the maintenance of a ‘non-differentiating trophoblast’ and their aberrant expression during early embryonic development, gestation, and parturition frequently leads to embryonic loss and the so-called abnormal offspring syndrome (AOS) [61].

Based on our results, Mel-LNC were more efficient than Mel-NC to improve the anti-apoptotic and antioxidant effects of melatonin during bovine embryo development. These findings suggest that the shape, architecture, surface chemical composition and component of the core could influence drug release and distribution in the culture medium in different ways. The melatonin-encapsulation efficiency was mainly influenced by the polymer nature. We showed in a previous study that melatonin formulations prepared with polymethacrylate (Eudragit^®^ S100) presented encapsulation efficiencies 56% while using polyester [poly(ε-caprolactone)] were around 39% and attributed this to the difference of melatonin affinity for these two polymeric materials [20, 39, 62]. Theoretically, in nanocapsules, drugs should be encapsulated by the polymer membrane and dissolved in the oil core, but it is known that some drugs are not completely water insoluble, which causes the drug to be dissolved in both the core and the aqueous suspension. The drug release mechanisms can be driven by the diffusion of the drug from the particles, the erosion/degradation of the particles, or both [38, 63].

Previously, we published studies using nanomaterials applied to sperm mediated gene transfer [27, 64]. Now, this is the first report using nanoencapsulated melatonin in embryo culture and the results obtained in this study might provide a promising strategy to supplement melatonin and other antioxidants, hormones and molecules poorly soluble in water, in culture media used for *in vitro* embryo production. Ongoing studies are being conducted by our research group to evaluate if Mel-LNC supplementation during oocyte *in vitro* maturation also provides advantages in relation to non-encapsulated melatonin.

The internalization mechanisms of NPs are only partially understood [60], furthermore it is important to clarify how the internalization of nanomaterials in gametes and embryos occurs, what happens to this material after fertilization and during embryo development, and what are the consequences of nanomaterial accumulation on fertility, embryo development and health of the offspring.

## Conclusions

In conclusion, we have shown that Mel-LNC is more efficient than Mel-NC, Mel, and the control groups to improve anti-apoptotic and antioxidant effects of melatonin during bovine embryo development. This work serve as a base for further studies that can increase the efficiency and durability of embryonic culture media, through supplementation and formulation of culture media with components carried in nanomaterials. In the future *in vivo* studies are planned.

## Supporting Information

S1 Table Primers sequences used for real-time PCR. (DOCX)

S2 Table *In vitro* development rates of bovine embryos cultured in SOFaa BSA media supplemented with free melatonin or nanocapsules loaded with melatonin. Proportion of bovine zygotes that cleaved and developed to 4, 8, 16-cell embryos, morulas and blastocysts.
(DOCX)

S3 Table Effect of non-encapsulated melatonin (Mel), melatonin-loaded in polymeric (Mel-NC) and lipid-core (Mel-LNC) nanocapsules on hatching blastocyst rate at Day 9. (DOCX)

S4 Table Effect of non-encapsulated melatonin (Mel), melatonin-loaded in polymeric (Mel-NC) and lipid-core (Mel-LNC) nanocapsules on cell number and apoptotic cell rate per blastocyst at D7. (DOCX)
