# Supplementary material for: Effects of Two Types of Melatonin-Loaded Nanocapsules with Distinct Supramolecular Structures: Polymeric (NC) and Lipid-Core Nanocapsules (LNC) on Bovine Embryo Culture Model
Source: PLoS One. 2016 Jun 16;11(6):e0157561. doi: 10.1371/journal.pone.0157561 (PMC4910990; doi:10.1371/journal.pone.0157561)
Supplement: S1 Table — (DOCX) [file pone.0157561.s001.docx]

Table 1. Primers sequences used for real-time PCR.

| **Primers** | **NCBI**  **Reference sequence** | **Sequence 5’→ 3’** | **Tm ºC** |
| --- | --- | --- | --- |
| *CASP*3 | NM_001077840.1 | F: GGACCCGTCAATTTGAAAAA | 55 |
|  |  | R: CATGTCATCCTCAGCACCAC | 55 |
| *BAX* | NM_173894.1 | F: CTACTTTGCCAGCAAACTGG | 56 |
|  |  | R: TCCCAAAGTAGGAGAGGA | 56 |
| *MCL1* | [NM_001099206.1](http://www.ncbi.nlm.nih.gov/entrez/viewer.fcgi?db=nucleotide&id=149642834) | F: TGTGGCCAAACACTTGAAGAGT | 60 |
|  |  | R: CCTTACGAGAACATCTGTGATGCTT | 60 |
| *NANOG* | NM_001025344.1 | F: TTCCCTCCTCCATGGATCTG | 58 |
|  |  | R: ATTTGCTGGAGACTGAGGTA | 58 |
| *SOX2* | NM_001105463.2 | F: CGAGTGGAAACTTTTGTCCG | 55 |
|  |  | R: GGTATTTATAATCCGGGTGTT | 55 |
| *OCT4* | [NM_174580.2](http://www.ncbi.nlm.nih.gov/entrez/viewer.fcgi?db=nucleotide&id=284055297) | F: GGTTCTCTTTGGAAAGGTGTTC | 55 |
|  |  | R: ACACTCGGACCACGTCTTTC | 55 |
| *GPX* | NM_174076.3 | F: GGACTACACCCAGATGAA | 60 |
|  |  | R: GTGGCGTCGTCACTTG | 60 |
| *SHC1* | [NM_001164061.1](http://www.ncbi.nlm.nih.gov/entrez/viewer.fcgi?db=nucleotide&id=255759964) | F: AAGTCAACGGGGACTTCCTT | 60 |
|  |  | R: GGCAAGTGATTGTCCATGTG | 60 |
| *CAT* | [NM_001035386.2](http://www.ncbi.nlm.nih.gov/entrez/viewer.fcgi?db=nucleotide&id=402693375) | F: GAACTGTCCCTACCGT | 56 |
|  |  | R: TCGTTGGCACTGTTGA | 56 |
| *PRDX5* | [NM_174749.2](http://www.ncbi.nlm.nih.gov/entrez/viewer.fcgi?db=nucleotide&id=31340842) | F: AGCCATGGCCCCGATTAAGG | 56 |
|  |  | R: AAGTGTTTATTGCAGAAATTTG | 56 |
| *SOD2* | [NM_201527.2](http://www.ncbi.nlm.nih.gov/entrez/viewer.fcgi?db=nucleotide&id=88853815) | F: CCCATGAAGCCTTTCTAATCCTG | 60 |
|  |  | R: TTCAGAGGCGCTACTATTTCCTTC | 60 |
| *β-actin* | NM_173979.3 | F: CTAGGCACCAGGGCGTCATG | 60 |
|  |  | R: CTTAGGGTTCAGGGGGGCCT | 60 |

F: forward; R: reverse
