# Supplementary material for: Effects of Two Types of Melatonin-Loaded Nanocapsules with Distinct Supramolecular Structures: Polymeric (NC) and Lipid-Core Nanocapsules (LNC) on Bovine Embryo Culture Model
Source: PLoS One. 2016 Jun 16;11(6):e0157561. doi: 10.1371/journal.pone.0157561 (PMC4910990; doi:10.1371/journal.pone.0157561)
Supplement: S2 Table — Proportion of bovine zygotes that cleaved and developed to 4, 8, 16-cell embryos, morulas and blastocysts. (DOCX) [file pone.0157561.s002.docx]

Table 2. *In vitro* development rates of bovine embryos cultured in SOFaa BSA media supplemented with free melatonin or nanocapsules loaded with melatonin.

Mel = Non-encapsulated melatonin, Mel-NC = melatonin-loaded polymeric nanocapsules, Mel-LNC = melatonin-loaded lipid-core nanocapsules, NC = drug-unloaded nanocapsules,, LNC = drug-unloaded lipid-core nanocapsules.

| Treatment | No of  oocytes cultured | No of  zygotes cultured | Cleavage rate  (%) | | No (%) of oocytes that developed to: | | | | |  |
| --- | --- | --- | --- | --- | --- | --- | --- | --- | --- | --- |
|  |  |  |  | 4-cell | | 8-cell | 16-cell | Mo | Blastocyst |  |
| Control | 192 | 165 | 141 (73) | 118 (61) | | 80 (42) | 72 (38) | 66 (34) | 63 (33) |  |
| MEL 10^-6^M | 175 | 158 | 128 (73) | 113 (65) | | 72 (41) | 66 (38) | 56 (32) | 48 (27) |  |
| MEL 10^-9^M | 176 | 154 | 124 (70) | 102 (58) | | 86 (49) | 75 (43) | 67 (38) | 58 (33) |  |
| MEL 10^-12^M | 158 | 141 | 117 (74) | 106 (67) | | 77 (49) | 73 (46) | 59 (37) | 52 (33) |  |
| MEL-NC 10^-6^M | 159 | 145 | 117(74) | 100 (63) | | 80 (50) | 77 (48) | 61 (38) | 54 (34) |  |
| MEL-NC 10^-9^M | 182 | 161 | 140 (77) | 118 (65) | | 89 (49) | 79 (43) | 71 (39) | 62 (34) |  |
| MEL-NC 10^-12^M | 176 | 158 | 132 (75) | 110 (63) | | 78 (44) | 65 (37) | 51 (29) | 42 (24) |  |
| NC (10^-6^M) | 181 | 158 | 126 (70) | 107 (59) | | 73 (40) | 69 (38) | 59 (33) | 52 (29) |  |
| MEL-LNC 10^-6^M | 176 | 158 | 132 (75) | 109 (62) | | 84 (48) | 75 (43) | 66 (38) | 63 (36) |  |
| MEL-LNC 10^-9^M | 176 | 150 | 120 (68) | 97 (55) | | 73 (41) | 72 (41) | 62 (35) | 52 (30) |  |
| MEL-LNC 10^-12^M | 158 | 139 | 119 (75) | 102 (65) | | 73 (46) | 72 (46) | 54 (34) | 42 (27) |  |
| LNC (10^-6^M) | 176 | 152 | 132 (75) | 119 (68) | | 81 (46) | 75 (43) | 61 (35) | 53 (30) |  |
