# Supplementary material for: Effects of Two Types of Melatonin-Loaded Nanocapsules with Distinct Supramolecular Structures: Polymeric (NC) and Lipid-Core Nanocapsules (LNC) on Bovine Embryo Culture Model
Source: PLoS One. 2016 Jun 16;11(6):e0157561. doi: 10.1371/journal.pone.0157561 (PMC4910990; doi:10.1371/journal.pone.0157561)
Supplement: S3 Table — (DOCX) [file pone.0157561.s003.docx]

Table 3S. Effect of non-encapsulated melatonin (Mel), melatonin-loaded in polymeric (Mel-NC) and lipid-core (Mel-LNC) nanocapsules on hatching blastocyst rate at Day 9.

| Treatment | Hatched blastocyst (%) D9 |
| --- | --- |
| Control | 8 (29.6)^a^ |
| MEL 10^-6^M | 16 (55)^b^ |
| MEL 10^-9^M | 17 (61)^b^ |
| MEL 10^-12^M | 18 (60)^b^ |
| MEL-NC 10^-6^M | 14 (61)^b^ |
| MEL-NC 10^-9^M | 15 (52)^b^ |
| MEL-NC 10^-12^M | 14 (52)^b^ |
| NC (10^-6^M) | 7 (25.9)^a^ |
| MEL-LNC 10^-6^M | 21 (70)^b^ |
| MEL-LNC 10^-9^M | 22 (91.7)^c^ |
| MEL-LNC 10^-12^M | 13 (65)^b^ |
| LNC (10^-6^M) | 8 (30.8)^a^ |

^a-c^Within the column, rates without a common superscript differed significantly (P < 0.05). Mel: non-encapsulated melatonin; Mel-NC: melatonin-loaded polymeric nanocapsules; Mel-LNC: melatonin-loaded lipid-core nanocapsules; LNC: Lipid-core nanocapsules.
