# Supplementary material for: Effects of Two Types of Melatonin-Loaded Nanocapsules with Distinct Supramolecular Structures: Polymeric (NC) and Lipid-Core Nanocapsules (LNC) on Bovine Embryo Culture Model
Source: PLoS One. 2016 Jun 16;11(6):e0157561. doi: 10.1371/journal.pone.0157561 (PMC4910990; doi:10.1371/journal.pone.0157561)
Supplement: S4 Table — (DOCX) [file pone.0157561.s004.docx]

Table 4S. Effect of non-encapsulated melatonin (Mel), melatonin-loaded in polymeric (Mel-NC) and lipid-core (Mel-LNC) nanocapsules on cell number and apoptotic cell rate per blastocyst at D7.

| Treatment | Number of  blatocysts  D7 | Total of number of nuclei  (mean ± SEM) | | Number of  TUNEL-stained nuclei  (mean ± SEM) | | | Apoptotic cell rate (%)  (mean ± SEM) |  |
| --- | --- | --- | --- | --- | --- | --- | --- | --- |
| Control | 16 | | 95.6 ± 6.7^a^ | | 10.7 ± 0.9^a^ | 10.1 ± 1,1^a^ | | |
| MEL | 13 | | 150.2 ± 20,3^b^ | | 8.5 ± 0.4^b^ | 5.6 ± 0.9^b^ | | |
| MEL-NC | 14 | | 133.3 ± 7.3^ab^ | | 11.2 ± 0.8^ab^ | 8.4 ± 1.1^ab^ | | |
| MEL-LNC | 14 | | 194.4 ± 5.5^c^ | | 3,8 ± 0.9^c^ | 1.9 ± 0.2^c^ | | |

^a-c^Within the column, rates without a common superscript differed significantly (P < 0.05). Mel: non-encapsulated melatonin; Mel-NC: melatonin-loaded polymeric nanocapsules; Mel-LNC: melatonin-loaded lipid-core nanocapsules.
